# Working Alliance and Subjective Engagement with a Digital Avatar CBT Platform (RITch^®^CBT): Comparing Young Adults with and Without Co-Occurring Substance Use and Depression

**DOI:** 10.3390/bs16050719

**Published:** 2026-05-07

**Authors:** Victoria Pezzino, Cassandra Berbary, Courtney McKinney, Celeste Sangiorgio, Emi Moriuchi, Korena S. Klimczak, Robert Kay Cooper, Wonkyung Kniffen, Maya Hareli, Cory Crane, Caroline J. Easton

**Affiliations:** 1 Division of Addiction Psychiatry and Research Program in Psychiatry, University of Rochester Medicine, Rochester, NY 14642, USA; cassandra_berbary@urmc.rochester.edu (C.B.); wonkyung_kniffen@urmc.rochester.edu (W.K.); cory_crane@urmc.rochester.edu (C.C.); caroline_easton@urmc.rochester.edu (C.J.E.); 2 Department of Clinical Health Professions, College of Health Sciences and Technology, Rochester Institute of Technology, Rochester, NY 14623, USA; cbmihst@rit.edu (C.M.); csihst@rit.edu (C.S.); mhareli@luriechildrens.org (M.H.); 3 Department of MIS, Marketing & Analytics, Saunders College of Business, Rochester Institute of Technology, Rochester, NY 14623, USA; emoriuchi@saunders.rit.edu; 4 Department of Psychology, Utah State University, Logan, UT 84322, USA; korena.klimczak@usu.edu; 5 Department of Psychology, University at Buffalo, State University of New York, Buffalo, NY 14203, USA; rkcooper@buffalo.edu; 6 Pritzker Department of Psychiatry and Behavioral Health, Ann & Robert H. Lurie Children’s Hospital of Chicago, Chicago, IL 60611, USA

**Keywords:** cognitive-behavioral therapy, working alliance, subjective engagement, substance misuse, depression, digital tools

## Abstract

Digital mental health interventions (DMHIs) can help bridge treatment gaps experienced by young adults with co-occurring substance misuse and depression. However, it remains unclear whether engagement with these interventions differs for young adults with co-occurring conditions compared to those experiencing substance misuse or depression alone. To investigate this issue, we assessed working alliance and subjective engagement with a digital avatar-assisted cognitive-behavioral therapy (CBT) treatment platform (RITch^®^CBT), comparing young adults with substance use, depression, and the co-occurrence of the two. A secondary data analysis was conducted on a sample of 99 young adults aged 18–28 years who presented at an urban university clinic. Participants rated their alliance and engagement following two brief sessions of the RITch^®^CBT platform. Participants were then categorized into behavioral health groups. Repeated exposure to the program had a greater impact on subjective engagement and usability across diagnostic conditions, but there was no difference in working alliance reported across sessions or behavioral health groups. Further, participants’ depressive symptoms were significantly correlated with the number of sessions they expressed they were willing to engage in and attend. Our findings suggest that digital tools may support early engagement in treatment for young adults, regardless of presenting problem.

## 1. Introduction

### 1.1. Young Adulthood and Substance Use

Young adulthood, defined as ages of 18 to 29, is a vital developmental stage during which individuals navigate identity amid frequent change and instability (Reifman & Niehuis, 2023; Walczak, 2023; Arnett et al., 2014; Schwartz et al., 2013). Due to these significant life experiences, young adults are more likely to experience mental health struggles and are at greater risk of developing psychiatric disorders compared to any other age group (Kessler et al., 2005; Substance Abuse and Mental Health Services Administration [SAMHSA], 2024; Williams et al., 2013). An inability to cope effectively, new autonomous roles, and frequent ambiguity are characteristic of this age range, which may result in increased experimentation and misuse of alcohol and drugs (McConaha et al., 2024; Silvers & Peris, 2023; Andrews & Westling, 2016). Though this increase can be considered normative, it can lead to chronic dependence, especially when risk factors such as negative family relationships, peer influence, externalizing behaviors, risk perception, increased stress, disinhibition, co-occurring mental health diagnoses, and exposure to violence and abuse are involved (Alhammad et al., 2022; Grigoriu & Benga, 2025; Sinha, 2024; Hill et al., 2000; Sheidow et al., 2012; Stone et al., 2012). Further, young adults are the most likely to use substances or report a substance use disorder in the past year compared to all other age groups (Substance Abuse and Mental Health Services Administration [SAMHSA], 2022; S. H. Adams et al., 2014).

Alcohol and cannabis use are consistently reported as the most prevalent substances among young adults and college-aged populations. Around 60% of college-aged students consume alcohol, and over a third engage in binge drinking (Z. W. Adams et al., 2025; American Addiction Centers [AAC], 2024; Martens, 2022). College students’ use of cannabis has also increased, with 2024 reports indicating the highest levels of use recorded among individuals aged 19 to 30 years old (Patrick et al., 2025). However, 2 out of 5 young adults also experiment with illicit drugs such as anti-anxiety drugs, opioids, and stimulants (American Addiction Centers [AAC], 2024). This experimentation makes young adults twice as likely to report an alcohol or drug use disorder in the past year compared to older adults (S. H. Adams et al., 2014; American Addiction Centers [AAC], 2024; National Institute on Drug Abuse [NIDA], 2023; Substance Abuse and Mental Health Services Administration [SAMHSA], 2025).

### 1.2. Substance Use, Misuse and Depression in Young Adulthood

Social norms, specifically on college campuses, often prevent young adults from recognizing or acknowledging substance use disorder or misuse, as the culture surrounding these campuses may promote binge drinking, permissiveness of misusing drugs for study strategies, and increased socialization around substance use (Gasa et al., 2022; Holm et al., 2022). Despite this, the effects of substance use can have detrimental effects on functioning and mood, contributing to internalizing and externalizing problems. Some of the most common reported mental health struggles among this population are depression, suicidal behaviors, and substance use (Goodwin et al., 2022; Hunt et al., 2020; Jones et al., 2023; Reinert et al., 2024; Viner & Tanner, 2009; Jurewicz, 2015). Substance use has been identified as a major risk factor for suicidal behavior among young adults, largely due to disinhibition and impulsivity (Borges et al., 2000; Jones et al., 2023). Individuals with depression may be especially vulnerable to developing substance use disorders because of maladaptive coping (Bondarchuk et al., 2025; Graupensperger et al., 2025; McGovern et al., 2023). Alternatively, chronic substance use can alter brain function and increase susceptibility to mood disorders in conjunction with external stressors during this developmental period (Dhami et al., 2022; Lijffijt et al., 2014; Iacono et al., 2008; Vik et al., 2004).

The prevalence of depression has increased, defined by a score greater than or equal to 10 on the Patient Health Questionnaire. Females report the highest rates of depression aged 12–19 years old, and 20–39 years old, followed by males aged 20 to 39 years old (Brody & Hughes, 2025). Roughly a quarter of adults aged 18–25 report a substance use disorder (SUD), with over 50% of young adults with SUDs reporting at least one co-occurring psychiatric disorder, though actual rates may be an underestimate due to self-reporting discrepancies and untreated cases (Silverstein et al., 2021; Steinhoff et al., 2023; Substance Abuse and Mental Health Services Administration [SAMHSA], 2024). The rising rates of both substance use and depressive symptoms among this age range place young adults at higher risk of developing co-occurring disorders, yet many may choose not to seek treatment (Lu et al., 2022). Identifying mental health problems and substance use is crucial during young adulthood, as the onset of lifetime psychiatric disorders tends to arise by age 30 with little increased rate of onset after (Kessler et al., 2005; Solmi et al., 2022).

### 1.3. Treatment Engagement Among Young Adult Populations

Though young adults may report more comorbid mental health and substance use compared to other age groups, they are also less likely to engage or adhere to treatment, with 9 in 10 young adults who report comorbid diagnoses not receiving treatment (S. H. Adams et al., 2014; Substance Abuse and Mental Health Services Administration [SAMHSA], 2025). Of those who do seek treatment, residential dropout rates are extremely variable and can extend up to 70% (Dillon et al., 2020; Substance Abuse and Mental Health Services Administration [SAMHSA], 2024). Treatment dropout may increase symptom severity and likelihood of developing psychiatric disorders, impair cognitive functioning, elevate risk of suicide or self-harming behaviors, and such individuals are more likely to experience early death (Hadland et al., 2021; Jones et al., 2023). Given high rates of depression and substance misuse, shared biological vulnerabilities, and low treatment engagement and high dropout among young adults, interventions that target co-occurring conditions and meet young adults where they are urgently needed; however, substantial barriers to care remain (Jones et al., 2023; Iacono et al., 2008). Common barriers include individual beliefs about treatment, such as stigma or that treatment is unnecessary; inadequate social supports; wait lists/times; cost of treatment; obtaining insurance approval; shortage of providers; legal barriers; and more (Farhoudian et al., 2022; Salaheddin & Mason, 2016). Many factors contribute to continued substance experimentation and reduced awareness of the harms and functional impacts of substance use among young adults. A common theme throughout literature is that the normative culture of substance use and potential stigma surrounding intervention and help from others drives use (Nordheim et al., 2018; Substance Abuse and Mental Health Services Administration [SAMHSA], 2019, 2024).

Factors that contribute to greater reductions in stress and increased engagement, attendance, and retention in treatment include young adult perceptions of higher working alliance and engagement in treatment (Andrade et al., 2019; Gidhagen et al., 2021; Munson et al., 2022; Urbanoski et al., 2012). There is a need for scalable, accessible, and effective interventions to promote engagement among this population. Prior research indicates that cognitive-behavioral therapy (CBT) is an effective intervention for various mental health problems across the globe. Specifically, CBT has been used as a target intervention for individuals with substance use and co-morbid mental health problems and has showed some benefit for individuals with alcohol or other drug use disorders and mental health disorders, and this intervention highly influences treatment retention for individuals who misuse alcohol or cannabis (Boness et al., 2023; Dalton et al., 2021; Magill et al., 2025; McHugh et al., 2010).

### 1.4. Avatar-Assisted Digital Treatment: An Innovative Approach

One mode that has proven effective in facilitating treatment among young adults is digital treatment, leveraging technology as a venue relatable to this age group and fostering control within their treatment environment (Carreiro et al., 2020; Hampton et al., 2024). The literature examining digital CBT demonstrates improvements in substance use, behavioral challenges such as aggression or conflict, and mood, especially when human components are involved using personalization and feedback components enhancing overall engagement and adherence (Easton et al., 2018a; Gilchrist et al., 2024; Moriuchi et al., 2023; Spek et al., 2007).

Cost-effective digitized mental health programs can help bridge treatment gaps for young adults with co-occurring substance use and depressive symptoms. However, it is unclear whether young adults with comorbid challenges, such as substance use and depression, engage differently with these platforms compared to young adults with one or fewer problems, such as substance use or depression alone, highlighting the need for more research with digital technology developments in this age range.

The study aims to evaluate young adults’ subjective engagement and working alliance with a digital avatar-assisted cognitive-behavioral therapy (CBT) treatment platform, RITch^®^CBT Platform, among substance-using young adults with depressive symptoms (Easton et al., 2018a, 2020; Moriuchi et al., 2023) Previous research has demonstrated a reduction in substance use and conflict using this platform, and patients report a preference for digital treatment compared to traditional approaches such as written materials (Easton et al., 2018a, 2018b; Moriuchi et al., 2023). It is hypothesized that young adults with comorbid challenges will report higher alliance and greater willingness to engage with this platform than those without comorbid challenges, due to the integrative nature of the platform’s approach to these presenting problems. An important goal of this study is to clarify how young adults with co-occurring substance use/misuse and depression interact with a brief two-session digital health intervention, in hopes of identifying mechanisms for refinement so the platform can be most effective for young adult populations who frequently present with comorbid substance misuse and mental health challenges.

## 2. Materials and Methods

### 2.1. Sample

A secondary data analysis from an original pilot study was completed. A total of 99 young adults (ages 18–28) were recruited from January to December 2024. Recruitment occurred via flyers and email “blasts” distributed around a large, northeastern, urban university clinic. Participants were invited to engage in a digital platform targeting co-occurring substance use and negative mood symptoms (e.g., aggression/conflict and depression). Eligible individuals were asked to complete two sessions using the RITch^®^CBT platform.

### 2.2. Study Design

In the original study, participants were randomly assigned to either the customization or non-customization group, in which they were either given the option to customize an avatar coach or a default avatar coach. The customization group was not the focus of the current investigation, so it was ignored in our analyses. Individuals could complete sessions in-person on the university campus or via a secure telehealth platform. At the first session, participants were provided a consent form, approved by Rochester Institute of Technology’s Institutional Review Board.

At session one, participants engaged with the platform for ~one hour. Participants reviewed the consent form, received an overview of the study, and were then assigned a login name by the research assistant to access the platform. Participants completed a variety of pre-intervention screening measures, including demographics and mental health screeners. Individuals then completed one module of the RITch^®^CBT Content Intervention and finished the session by answering standardized post-intervention measures. About one week later, they completed a second module of the RITch^®^CBT platform and answered the same post-intervention measures. See below for session content. Each participant was provided a $50 gift card for each completed session ($100 total).

### 2.3. Measures

#### 2.3.1. Pre-Intervention Screening Measures

Participants completed pre-intervention questionnaires that asked about demographics, current substances used, medical conditions, and mental health conditions.

##### RITch^®^CBT Intervention

RITCH Additional Questions. Before engaging in the RITch^®^CBT content, participants could customize their avatar coach’s gender, hair color, haircut, tone of voice, clothing style, etc. Participants then answered additional questions to assess engagement and alliance with the platform, including whether they practiced the coping skills taught and whether those skills were perceived as helpful. Ratings were provided on a 0 to 10 scale, with participants, on average, reporting at session 2 a mean of 3.10 (SD = 2.50) regarding whether they practiced the skills and 4.13 (SD = 2.84) for how helpful they found the skills to be.

RITch^®^CBT platform. RITch^®^CBT is a manualized CBT intervention that simultaneously targets substance use, aggression/conflict, communication skills, and negative mood symptoms such as depression. This program is a digitized, fully interactive platform that allows the participants to self-guide with the assistance of an avatar coach. The avatar coach narrates the material in a consistent and standardized manner to administer CBT. Each session is 1:1 and covers content specific to the module chosen. As the avatar narrates the content, the participant follows along (either on an iPad or computer). In the modules, participants will use slider scales to answer session material, assessment and coping skill exercises. At different intervals during the session, the avatar will ask the participants to rate their own unique experiences on their tablets. Participants engage with the platform for 30–60 min, depending on how quickly or slowly they may answer the material. The session content is broken up into three segments, where in the first half of the module, psychoeducation is provided on the topic area, the second part of the module introduces new material or coping strategies, and the third part of the module requires participants to practice skills taught and to reflect on when they can use these strategies.

This manualized treatment has previously been studied among populations to reduce substance use and conflict with others (Easton et al., 2018a, 2018b; Moriuchi et al., 2023). Due to the pilot nature of this study, only two modules were covered, focusing on psychoeducation surrounding substance and conflict, increasing self-awareness, and introducing alternative coping skills. These modules were covered across the two sessions and included: (1) understanding patterns of substance use and conflict, and (2) identifying triggers for substance use, negative mood symptoms, and conflict. While using this intervention, participants were required to be actively engaged by responding to prompts and completing exercises, as the program does not move forward unless these items are completed. Research assistants were present in case any technological problems arose. Specific avatar features that may help with engagement include corrective feedback for responses, confetti bursts, awards (ribbons/trophies) participants could earn, and the potential to customize an avatar coach versus using a default avatar coach. It is important to note the potential influence customization may have had on the results of this study. Participants who could customize their avatar coach may perceive the platform as more usable and engaging due to more opportunities for personalization features. Despite this, all participants were exposed to the gamified feedback and positive reinforcement features.

#### 2.3.2. Post-Intervention Measures

Mental Health Screeners. All participants were provided post-intervention self-report questionnaires to assess mental health, substance use, and perceived engagement with the platform. Substance Use. Each participant completed the Timeline Follow-Back method (TLFB; Agrawal et al., 2009; McCann et al., 2025) to collect the last seven-day substance use (i.e., alcohol, cocaine, cannabis, stimulants, opioids, heroin, hallucinogens, sedatives, benzodiazepines, inhalants, and other). The most commonly occurring substance endorsements were alcohol and cannabis, which is consistent with the previous literature; these substances were examined further over time. A composite score was created that represents the total number of days of substance use for each endorsed substance. This composite score was then dichotomized into a single substance use score for alcohol and cannabis and a total substance use score to distinguish between participants who did not use any substances and those who reported use in the past seven days. Those who reported having used any substance within the past seven days on the TLFB were coded as endorsing substance use. TLFB was completed at both sessions 1 and 2. Depression. Participants completed the Patient Health Questionnaire (PHQ-9) at the end of each session to assess the severity of depressive symptoms reported. Each participant rated on a 4-point Likert scale how often they experienced each symptom (0 = not at all, 3 = nearly every day). The total score was obtained by summing the scores for each question, which ranged from 0 to 27, with higher scores indicating more severe depression (Kroenke et al., 2001; Pitts et al., 2023). Participants who reported ≥ 10 on the PHQ-9, indicating moderate depressive symptoms, were coded as endorsing depressive symptoms.

Engagement and Alliance Questionnaires. Engagement and Alliance Measures: Adapted to reference Avatar/Therapist instead of “Application and Therapist.” To further assess participants’ responses, evaluations of engagement and alliance with the platform were completed. User Engagement. To assess engagement, participants completed the User Engagement Scale—Short Form (UES-SF) after each session of the digital platform (Lalmas et al., 2022; Moilanen et al., 2022; O’Brien et al., 2018). Participants rated their engagement with the digital tool on a 5-point Likert scale (1 = strongly disagree, 5 = strongly agree), with four subscales (focus, usability, aesthetic, and reward). These subscales are used to assess the quality of user experience with key components of assessing engagement as it relates to one’s ability to attend to the information presented to them and novelty of the program, participant perceptions of the program as easy to use, appearance and appeal towards the program, and if the participant experiences felt involvement or finds the program interesting (Lipschitz et al., 2023; O’Brien et al., 2018). Mean scores for the individual subscales and an overall score were computed at session 1 and session 2 by averaging items within each subscale and the total. Usability items were reverse-scored, with higher scores indicating greater usability (Lalmas et al., 2022; Moilanen et al., 2022; O’Brien et al., 2018).

Working Alliance. The working alliance between participants and the digital platform was assessed at each session using the Brief-Revised Working Alliance Inventory (BR-WAI; Bilek et al., 2026; Mallinckrodt & Tekie, 2015; van Benthem et al., 2024). The original version of the BR-WAI includes 16 items ranging from 16 to 80. Our study utilized a modified version that included 6 items, ranging from 6 to 30 and included the following questions: “As a result of these sessions, I am clearer as to how I might be able to change”, “What I am doing in therapy gives me new ways of looking at my problem”, “The avatar and I collaborate on setting goals for my therapy”, “ The avatar and I are working towards mutually agreed upon goals”, “I feel that the things I do in therapy will help me to accomplish the changes that I want”, and “ I believe the way we are working with my problem is correct.” These items were scored on a 5-point Likert scale (1 = seldom, 5 = always). A total score was obtained by summing the results. Higher scores indicate a stronger alliance, though there is no specific cutoff. The scale was shortened in the primary study to improve construct validity, as participants could not engage in specific conversations or disagreements with the platform. The 6-item version used was found to be highly reliable (α = .88). Additional Questions. At the end of each session, participants were asked, “How many sessions would you complete using the RITch^®^CBT Platform?” to assess perceived engagement. Participants could rate on a scale of 0 to 10.

### 2.4. Data Analysis

Descriptive statistics were tabulated for demographics, substance use characteristics, and mental health symptoms reported within the sample, including means, standard deviations, and percentages. Statistical significance was determined for all analyses using an alpha level of 0.05. Based on the TLFB measure of substance use and the PHQ-9 of reported depressive symptoms, participants were categorized into behavioral health (BEH) groups. These variables were summed to provide a baseline score at session 1 and then dichotomized. If participants endorsed any substance use in the past 7 days and depressive symptoms below 10, they were assigned a 1 and categorized into the substance use only BEH group, and if they endorsed no substance use and a score of 10> and above on the PHQ-9, indicating moderate depressive symptoms present, they were assigned a 1 and categorized in the depression-only BEH group. Participants who did not endorse any substance use and a PHQ-9 score below 10 were assigned to the no-substance-use-or-depression BEH group, while participants who endorsed both substance use and moderate depressive symptoms were assigned to the co-occurring BEH group. To preliminarily investigate the program’s efficacy, a series of paired samples *t*-tests was used to assess changes in substance use and depressive symptoms from session one to session two for the overall sample.

Further, a series of repeated-measures ANOVAs was conducted of the 91 participants who completed both session 1 and session 2 of the platform to assess within- and between-subject effects for each behavioral health group’s ratings of engagement, working alliance, and the number of sessions they would hypothetically like to complete in the future. Given the limitations of reducing depression and substance use to discrete categories, we also assessed these variables continuously. Specifically, we conducted linear regression to assess whether the severity of depressive symptoms or the number of days of substance use predicted engagement and working alliance. A separate model was run for engagement and working alliance, respectively, in which both depression and days of substance use were used as predictors in each model. We also tested whether there was a correlation between depressive symptoms and the number of sessions they would like to complete from participants who completed both sessions 1 and 2. To assess perceived helpfulness and participants’ use of skills, a series of one-way ANOVAs was used to determine differences between behavioral health groups, which included the 91 participants who completed session 2 and rated this scale within the platform. Missing data were coded as missing values and excluded from analyses using listwise deletion. Analyses included all non-missing data for each model. All analyses were conducted with IBM SPSS Statistics Version 31.0 (IBM Corp., 2024; Armonk, NY, USA) and with R (v4.5.2; R Core Team, 2026) and RStudio (v 2026.04.0+526; Posit Team, 2025).

## 3. Results

### 3.1. Participant Characteristics

Demographics, substance use, and mental health characteristics are shown in Table 1. Participants included 99 young adults aged 18–28 years old (M = 20.9, SD = 1.81). The majority completed high school (33.3%) and some college (39.4%), and indicated their racial background as Caucasian (68.7%), followed by Asian (22.2%), Black (4%), or Latino/a (5.1%). More than half of the sample identified as women (56.6%), and approximately one-third self-reported one or more mental health conditions (39%) at baseline. The following diagnoses were reported at baseline: obsessive–compulsive disorder (7.1%), anxiety (35.4%), attention-deficit hyperactivity disorder (11.1%), depression (19.2%), post-traumatic stress disorder (3%), autism disorder (2%), panic disorder (1%), and other diagnoses (3%). Of note, these were self-reported diagnoses, and due to potential misclassification, we assigned groups to their respective behavioral health groups based on standardized screening measures (PHQ-9, TLFB) instead of self-report.

A total of 17.2% (*n* = 17) of participants endorsed substance use and depression symptoms, 34.3% (*n* = 34) endorsed substance use only, 12.1% (*n* = 12) depressive symptoms only, and 36.4% (*n* = 36) endorsed neither substance use nor moderate depressive symptoms. A total of 91 young adults returned for a second session of the brief intervention and completed all corresponding questionnaires. Given that this sample was taken from a general college campus and not a treatment center or outpatient clinic, it is typical for the symptoms participants are most frequently reporting to include substance use only or no significant depression or substance use. This is consistent with the prior literature, where 22 to 49.6% of students on college campuses have used substances within the past month (Substance Abuse and Mental Health Services Administration [SAMHSA], 2023). Therefore, while interpreting these findings, it is important to keep in mind that trends of alliance and engagement may vary with larger sample sizes in the depression only or co-occurring group, and if this intervention was used in a treatment setting with participants who may be more motivated to receive treatment and are experiencing more significant impairment in their daily lives. As a result, these findings may generally reflect engagement and alliance experiences of young adults who report less complex clinical presentations, though still reporting moderate depression and co-occurring substance use.

### 3.2. Preliminary Analyses

#### Substance Use and Depression Across Sessions

Before engaging with the CBT digital platform, 57.6% (*n* = 57) of participants reported using a substance at least once within the past week, on average using 3.55 times (*SD* = 6.34). Substances participants reported using included alcohol (50.5%, *n* = 50), cannabis (23.2%, *n* = 22), stimulants (6.4%, *n* = 6), hallucinogens (2.2%, *n* = 2), sedatives (1.1%, *n* = 1), and other (5.1%, *n* = 4). There was no reported use of cocaine, crack, opioids, heroin, benzodiazepines, or inhalants. In our analyses, we examined the most frequently used substances, alcohol and cannabis. Specifically, the frequency of alcohol use reduced from session one (*M* = 0.82, *SD* = 1.15, 95% CI [0.57, 1.06]) to session two (*M* = 0.53, *SD* = 1.01, 95% CI [0.32, 0.74], *t*(90) = 3.44, *p* < .001, 95% CI [0.12, 0.45], η^2^*_p_* = .12). No other significant reductions from session 1 to session 2 were found for other substances (all *p*s > .05). Depressive symptoms reported on the PHQ-9 did not significantly differ from session one (*M* = 6.54, *SD* = 4.82, 95% CI [5.54, 7.54]) to session two (*M* = 6.30, *SD* = 4.83, 95% CI [5.29, 7.30]; *t*(90) = 0.59, *p* = .555, 95% CI [0.57, 1.05], η^2^*_p_* = .004). See Table 2. 

### 3.3. Engagement and Alliance Outcomes

#### 3.3.1. Engagement

A four-group (BEH) × 2 (time) repeated-measures ANOVA was analyzed to assess differences in self-reported subjective engagement (i.e., UES-SF) between BEH groups, and if there were changes across sessions (see Table 2). There was a significant main effect of time on total subjective engagement (*F*[1, 87] = 11.72, *p* < .001, *η*^2^*_p_* = .12), which increased from session one (*M* = 3.00, *SD* = 0.63, 95% CI [2.87, 3.13]) to session two (*M* = 3.23, *SD* = 0.67, 95% CI [3.09, 3.37]). There was no main effect of the BEH group (*F*[3, 87] = 0.663, *p* = .577, *η*^2^*_p_* = .02), nor a time × BEH interaction (*F*[3, 87] = 1.47, *p* = .228, *η*^2^*_p_* = .05). See Table 3.

#### 3.3.2. Engagement Subscales

Aesthetic. While there was no main effects of time (*F*[1, 87] = 0.03, *p* = .858, *η*^2^*_p_* < .001) and BEH (*F*[3, 87] = 1.33, *p* = .271, *η*^2^*_p_* = .04) for the UES-SF aesthetic subscale, there was a significant time × BEH interaction (*F*[3, 87] = 3.37, *p* = .022, *η*^2^*_p_* = .10). Follow-up analyses using pairwise comparisons within each group showed that the no substance use and depression and co-occurring groups did not change significantly between sessions. The results indicated that the interaction was driven by opposing patterns of change in the substance use and depressed only groups. The substance use only group exhibited an increase in aesthetic score from session one (*M* = 2.07, *SD* = 0.83, 95% CI [1.78, 2.36]) to session two (*M* = 2.33, *SD* = 0.77, 95% CI [2.06, 2.61]; *p* = .039), while the depressed only group exhibited a decrease in aesthetic score from session one (*M* = 2.88, *SD* = 1.00, 95% CI [2.20, 3.55]) to session two (*M* = 2.36, *SD* = 0.95, 95% CI [1.73, 3.00]; *p* = .020). Usability. As for the UES-SF usability subscale, there was a significant main effect of time (*F*[1, 87] = 4.41, *p* = .039, *η*^2^*_p_* = .05), as all participants rated the platform as more usable during session two (*M* = 4.31, *SD* = 0.75, 95% CI [4.16, 4.47]) than session one (*M* = 4.14, *SD* = 0.87, 95% CI [3.96, 4.32]). There was no main effect of a BEH group (*F*[3, 87] = 0.94, *p* = .423, *η*^2^*_p_* = .03), nor a time × BEH interaction (*F*[3, 87] = 0.66, *p* = .581, *η*^2^*_p_* = .02). Reward. As for the UES-SF reward subscale, there was no significant main effect of time (*F*[1, 87] = 0.25, *p* = .617, *η*^2^*_p_* = .003), of a BEH group (*F*[3, 87] = 0.08, *p* = .970, *η*^2^*_p_* = .003), nor a time × BEH interaction (*F*[3, 87] = 0.24, *p* = .865, *η*^2^*_p_* = .008). Focus. As for the UES-SF focus subscale, there was no significant main effect of time (*F*[1, 87] = 0.03, *p* = .861, *η*^2^*_p_* < .001), a BEH group (*F*[3, 87] = 1.24, *p* = .299, *η*^2^*_p_* = .04), nor a time × BEH interaction (*F*[3, 87] = 0.54, *p* = .658, *η*^2^*_p_* = .02).

#### 3.3.3. Working Alliance

A four-group (BEH) × 2 (time) repeated-measures ANOVA was analyzed to assess behavioral health groups' working alliance changes over time. The tests revealed no significant effects of time, (*F*[1, 87] = 0.72, *p* = .398, *η*^2^*_p_* = .01), nor time × BEH group interactions, (*F*[3, 87] = 0.54, *p* = .656, *η*^2^*_p_* = .02), nor main effect of BEH group, (*F*[3, 87] = 0.66, *p* = .579, *η*^2^*_p_* = .02), though there was a general trend upward where young adults reported higher alliance at session two (*M* = 16.56, *SD* = 5.21, 95% CI [15.47, 17.65]) compared to session one (*M* = 15.80, *SD* = 5.84, 95% CI [14.59, 17.02]) across diagnostic conditions. This is consistent with literature indicating observed working alliance differences are generally not detected until three or more sessions (Hersoug et al., 2001; Mallinckrodt & Tekie, 2015).

#### 3.3.4. Additional Questions-Number of Sessions

Participants were asked to assess the number of sessions they would be willing to engage with the RITch^®^CBT platform. Overall, participants rated they would engage with the platform for approximately four sessions at session 1 (*M* = 4.39, *SD* = 2.9, 95% CI [3.81, 4.97]) and session 2 (*M* = 4.27, *SD* = 2.94, 95% CI [3.66, 4.89]), with majority of participants reporting they would prefer a briefer intervention (four or less sessions) and over one-third of young adults reporting they would use the platform for 5 to 10 sessions. To assess whether there was a difference between BEH groups and preference for sessions they were willing to engage in, a repeated ANOVA was again conducted. No significant differences were observed across diagnostic conditions for time (*F*[1, 87] = 0.35, *p* = .554, *η*^2^*_p_* = .004), nor time × BEH group (*F*[3, 87] = 0.42, *p* = .740, *η*^2^*_p_* = .01), nor the main effect of the BEH group (*F*[3, 87] = 0.79, *p* = .505, *η*^2^*_p_* = .03).

While the number of sessions participants would engage in did not differ by BEH, there was a significant positive correlation between the severity of depression symptoms and how many sessions participants endorsed they would engage with the RITch^®^CBT platform (r_2_ = 0.22, *p* = .031). There was no association between the severity of total substance use and how many sessions participants endorsed they would engage with the RITch^®^CBT platform (r_2_ = −0.001, *p* = .989).

### 3.4. Severity of Symptoms Analyses

#### 3.4.1. Number of Sessions and Self-Reported Symptoms

To better understand how the severity of depression and substance use relates to engagement and working alliance, we analyzed these variables continuously using linear regression. Neither depression symptoms (*β* = 0.02, *SE* = 0.01, *p* = .157, 95% CI [−0.01, 0.04]) nor days of substance use (*β* = −0.02, *SE* = 0.03, *p* = .619, 95% CI [−0.08, 0.05]) predicted subjective engagement; *F*(2, 96) = 1.03, *p* = .360, *R*^2^ = .021. Similarly, neither depression symptoms (*β* = 0.12, *SE* = 0.13, *p* = .363, 95% CI [−0.14, 0.37]) or days of substance use (*β* = 0.01, *SE* = 0.31, *p* = .985, 95% CI [−0.60, 0.61]) predicted working alliance; *F*(2, 96) = 0.44, *p* = .644, *R*^2^ = .01. Additionally, neither depression (*β* = 0.07, *SE* = 0.07, *p* = .273, 95% CI [−0.06, 0.20]) or days of substance use (*β* = 0.07, *SE* = 0.16, *p* = .667, 95% CI [−0.25, 0.39]) predicted the number of sessions participants would have hypothetically liked to continue completing; *F*(2, 88) = 0.84, *p* = .434, *R*^2^ = .012.

#### 3.4.2. RITch^®^CBT Platform Engagement and Alliance Questions

A series of one-way ANOVAs was conducted to examine whether there was a difference between diagnostic groups and their ratings of coping skill use and perceived helpfulness using the platform. There were no observed differences between BEH groups and their ratings of coping skill practice (*F*[3, 62] = 1.91, *p* = .138, *η*^2^*_p_* = .08), as well as their ratings of perceived helpfulness (*F*[3, 62] = 1.39, *p* = .255, *η*^2^*_p_* = .06). For this analysis, the variable level missingness exceeded 5%, though it is likely that the data is missing completely at random due to participants using the app for the first time and potential technical or wifi issues that could arise during initial use. As such, due to the randomness of missingness, it is not likely to bias these results.

## 4. Discussion

This study sought to investigate how depressive symptoms, substance use, and their co-occurrence related to subjective user engagement with a self-guided online platform called RITch^®^CBT. RITch^®^CBT is an online program intended to simultaneously treat substance use, aggression and conflict, communication skills, and negative mood symptoms such as depression. Participants completed up to two sessions of the program a week apart. The skills covered included identifying triggers for substance use and aggression, planning for distractions to use when experiencing triggers, improving awareness of warning signs of anger, and using coping skills for anger (e.g., counting to 10, drinking a cool glass of water, taking a moment to sit and relax, etc.).

Prior research has demonstrated that individuals with greater levels of psychopathology are more likely to express an interest in using digital mental health programs, despite these symptoms providing barriers to continued usage of online programs in practice (Borghouts et al., 2021; Borgnolo et al., 2025). Additionally, the more tailored and relevant a program is to a user’s needs, the more likely engagement is to occur (Pardini et al., 2022; Saleem et al., 2021; Wanniarachchi et al., 2025). Thus, we hypothesized that participants who use substances and experience moderate to severe depressive symptoms concurrently would experience greater levels of subjective engagement and perceived working alliance. This was not necessarily supported by our findings, which suggest that substance use and depression symptoms, as well as their co-occurrence, have no significant relationship to subjective engagement or working alliance. This was the case both when analyzing these variables categorically (e.g., comparing those with at least one day of substance use in the past week, at least moderate depressive symptoms, co-occurring use, and neither) as well as continuously as a measure of severity. While neither predicted how many sessions they would be hypothetically interested in completing in the future, a significant positive correlation was found between this and depression.

Repeated exposure to the program appeared to have a greater impact on subjective engagement than depression or substance use, as engagement significantly increased from session 1 to session 2. When following up to see whether this effect was specific to certain subscales of engagement, we found that, in particular, perceived usability (e.g., whether the program was easy to use) significantly improved between sessions, with no changes in focused attention (e.g., how immersed they were in the program), aesthetic elements (e.g., how appealing the graphics were), or reward factor (e.g., whether the program was interesting and worthwhile) (Lalmas et al., 2022; Lipschitz et al., 2023; O’Brien et al., 2018).

We additionally found that individuals who used substances reported no depressive symptoms were significantly more likely to rate aesthetic elements higher in session 2 compared to session 1, while the reverse was found for those with depressive symptoms and no substance use. It is possible that those in the substance use group were more receptive to the program and, by extension, its visual presentation. While this would make sense given the program’s focus on substance use reduction, with no content explicitly focusing on depression, this interpretation should be taken lightly, as the effect did not extend to other elements of engagement and working alliance. Additionally, this contradicts the prior literature, which establishes that aesthetic appeal makes users more receptive to programs vs. the other way around (Denison-Day et al., 2023; Sonderegger & Sauer, 2010).

Overall, engagement and alliance with the program appeared to be rated relatively neutral on average, falling in the “neither agree nor disagree” range for the former and between “disagree” and “neutral” for the latter. Regardless, participants demonstrated interest in hypothetically completing more sessions of the program. While a randomized controlled trial is called for to establish the efficacy of the program, we found a significant reduction in alcohol use on average. Our study may provide preliminary evidence that young adults can be open to completing a substance use-focused program regardless of their own use status and depression psychopathology.

It is well established that it is difficult to have individuals behaviorally engage in programs, as rates of uptake and completion for publicly available programs are generally low (Lipschitz et al., 2023). However, our findings may provide support for initiatives that build online interventions into existing programs, thus encouraging engagement. For example, colleges may include substance use prevention programming, with an increasing number including brief interventions for mental health and implementing screenings to identify students at risk of substance misuse (Babor et al., 2008; Committee on Mental Health, Substance Use, and Wellbeing in STEMM Undergraduate and Graduate Education et al., 2021; Vereschagin et al., 2024). Despite not being directly relevant to all who may complete the program, our findings suggest that this does not necessarily detract from individuals’ ability to connect with and attend to the program. This supports the implementation of substance use and mental health-focused programs in contexts that cast a wide net (e.g., clinic lobbies, a standard resource for those on a behavioral health waitlist, etc.), with some confidence that this will not produce an iatrogenic effect such as dissatisfaction that could dissuade future engagement with similar programs.

Our findings both aligned and diverged from adjacent research in a few different ways. It is well established that lack of fit between a digital intervention’s content and an individual’s specific needs is typically a barrier to engagement (Lipschitz et al., 2023; Oesterle & Bormann, 2026). Given that RITch^®^CBT specifically targets substance use, it is unclear why those who did not use any substances reported similar levels of engagement and alliance to those who did. One possibility is that RITch^®^CBT may not have engaged the target population of substance users enough to detect a difference from non-users. Indeed, the neutral levels of working alliance reported were lower than those of other digital substance use interventions, in which alliance was similar to clinician-delivered CBT (Benitez et al., 2023).

Looking at substance use and depression more generally, regardless of considerations towards fit, lower severity of these characteristics have been found to be predictive of uptake (e.g., beginning to use a program) but not of ongoing usage (Zainal et al., 2026). This finding regarding usage was replicated in a large trial on a digital intervention specific to alcohol use (Crawford et al., 2024) and real-world engagement data for a digital substance use program (Günther et al., 2023). Similarly, the severity of depression and substance use appeared to be unrelated to engagement in our study.

It is less clear how the improvements in subjective engagement from session 1 to session 2 compare to prior research. Subjective engagement and working alliance are typically measured cross-sectionally at post-intervention assessment, leaving the relationship between attitudes towards programs and increased exposure to the intervention unclear. Looking beyond clinical intervention research, attitudes towards a given technology have been shown to be dynamic, particularly in the early stages of program engagement (Bhattacherjee & Premkumar, 2004). This shows promise for work in this area despite the current paucity of related studies. The increase in subjective engagement from session 1 to session 2 has important implications for digital interventions targeting substance use. Prior work has found that increases in digital alliance over time are predictive of decreases in alcohol use (Benitez et al., 2023). While a change in alliance was absent in the present study, subjective engagement is highly related to alliance and similarly predicts improvements in treatment outcomes (Graham et al., 2021).

Participants expressing an interest in completing more sessions is promising, with prior work finding that completing more sessions of a digital substance use program improves the odds of abstinence (Luderer et al., 2022). However, this finding should be treated as preliminary, as expressed interest in a program is often not necessarily predictive of actual follow-through (Bowers et al., 2026). Regardless, brief interventions still hold value, demonstrated by the reduction in alcohol use found in the present study, with similar small effects found in other brief single-session interventions (Boumparis et al., 2019; Ghosh et al., 2022). The lack of significant change in depressive symptoms may be attributable to the lack of depression-focused content in the intervention. Meta-analyses have indicated that substance use-focused interventions often have an effect on depression, but these effects are not necessarily consistent across studies, and work in this area is still emerging (Magill et al., 2023, 2025).

Overall, our study provides preliminary evidence that young adults can be receptive to digital substance use interventions such as RITch^®^CBT, regardless of co-morbidity status. Such intervention can be helpful for reducing substance use, including in brief formats such as the intervention provided in this study (Boumparis et al., 2019; Ghosh et al., 2022). Subjective engagement, particularly usability, appears to be a dynamic construct that can improve with continued usage. More work is necessary to clarify if this is also true for working alliance, as no significant changes between sessions were found in our study.

### Limitations and Future Research

Our findings should be evaluated within the context of several limitations. First, we used a relatively small sample of under 100, making it unclear if the analyses were underpowered (Lakens, 2022). The primary analyses categorized participants into four groups with relatively uneven representation; thus, our sample size may have been too small to detect differences. It is also unclear whether our findings will appropriately generalize to Black, Latino/a, or Native American/Indigenous populations, given that our sample primarily consisted of White and Asian individuals. Recruiting a more diverse sample in the future is called for.

Additionally, our measure of substance use may be flawed. The number of days of use does not necessarily directly map onto the severity of problematic substance use, both in terms of quantity consumed, as well as psychological symptoms associated with substance use disorder (Volkow & Blanco, 2023). By only measuring substance use within the past seven days to bucket participants as users versus non-users, the use captured may not represent participants’ longer-standing relationship with substances (i.e., participants could have been heavy users before the seven days or be an infrequent user but happened to use during the period of the study). As a result, it is somewhat unclear if our co-occurring group was conceptually distinct from the depression and substance use only groups. It is also unclear how aggression factors into how well participants connected with the program, given that intervening on aggressive behavior was a key component of the skills presented, yet this was not measured (Smith et al., 2025).

Additionally, this is the first research study published examining the app’s engagement with young adults. It is unclear whether the data that was missing is a constant theme in other trials using this platform, but it is likely to be missing at random due to first-time use and potential technical or connectivity issues that could arise using digital tools. Further research is necessary to better understand the relationship between substance use, depression, program relevance, how programs are received, and their efficacy. It remains unclear whether co-occurring substance use and depression are distinct from each individual issue in how it interacts with program engagement and acceptability, as this is the first study to investigate this issue (Jonathan et al., 2025).

A future randomized controlled trial will be necessary to better understand the relationship between subjective engagement, working alliance, and RITch^®^CBT’s efficacy. Brief interventions are an important area of work; however, evaluating outcomes further out than one week will be critical for understanding longer-term impact. This study only included the first 2 sessions of RITch^®^CBT, which contains 14 sessions in total. Further investigation with a longer treatment window can provide insight into the dose–response relationship, and the additive effect of completing additional sessions on treatment efficacy. Future work can shed light on what interventions and participant characteristics shape engagement, allowing us to better understand underlying mechanisms.

## Figures and Tables

**Table 1:** Baseline participant demographic characteristics at session 1.

Characteristics	Total *N* = 99	Depression Only *N* = 12	Substance Use Only *N* = 34	Co-Occurring *N* = 18	No Substance Use or Depression *N* = 36	
			Mean (SD), Range *n* (%)			χ^2^ (*p*) *F* (*p*)
Age, in years	20.92 (1.81), 18–28	21.00 (1.76), 19–24	20.91 (1.44), 18–25	21.53 (2.10), 18–25	20.60 (2.00), 18–28	1.01 (.392)
Race						
White	68 (68.7%)	7 (58.3%)	27 (79.4%)	14 (82.4%)	20 (55.6%)	12.03 (.211)
Asian	22 (22.2%)	4 (33.3%)	5 (14.7%)	1 (5.9%)	12 (33.3%)	
Black	4 (4.0%)	0 (0.0%)	0 (0.0%)	1 (5.9%)	3 (8.3%)	
Latin	5 (5.1%)	1 (8.3%)	2 (5.9%)	1 (5.9%)	1 (2.8%)	
Gender						
Woman	56 (56.6%)	9 (75.0%)	22 (64.7%)	8 (47.1%)	17 (47.2%)	13.64 (.034)
Man	39 (39.4%)	1 (8.3%)	11 (32.4%)	8 (47.1%)	19 (52.8%)	
Non-Binary	4 (4.0%)	2 (16.7%)	1 (2.9%)	1 (5.9%)	0 (0.0%)	
Education						
High School	33 (33.3%)	3 (25.0%)	8 (23.5%)	7 (41.2%)	15 (41.7%)	9.55 (.655)
Some College	39 (39.4%)	7 (58.3%)	16 (47.1%)	7 (41.2%)	9 (25.0%)	
Associates	4 (4.0%)	1 (8.3%)	1 (2.9%)	0 (0.0%)	2 (5.6%)	
Bachelors	17 (17.2%)	1 (8.3%)	7 (20.6%)	2 (11.8%)	7 (19.4%)	
Masters	6 (6.1%)	0 (0.0%)	2 (5.9%)	1 (5.9%)	3 (8.3%)	
Self-Report Mental Health Conditions						
Total Reported	0.78 (1.15), 0–4	1.33 (1.30), 0–4	0.76 (1.10), 0–3	1.23 (1.48), 0–4	0.42 (0.81), 0–3	3.25 (.025)
Depression	19 (19.2%)	5 (41.7%)	6 (17.6%)	4 (23.5%)	4 (11.1%)	5.68 (.128)
Anxiety	35 (35.4%)	8 (66.7%)	11 (32.4%)	8 (47.1%)	8 (22.2%)	9.02 (.029)
OCD	7 (7.1%)	1 (8.3%)	3 (8.8%)	1 (5.9%)	2 (5.6%)	0.35 (.950)
PTSD	3 (3.0%)	1 (8.3%)	1 (2.9%)	1 (5.9%)	0 (0.0%)	2.75 (.433)
Panic	1 (1.0%)	0 (0.0%)	0 (0.0%)	1 (5.9%)	0 (0.0%)	4.87 (.181)
Autism	2 (2.0%)	0 (0.0%)	0 (0.0%)	2 (11.8%)	0 (0.0%)	9.85 (.020)
ADHD	11 (11.1%)	1 (8.3%)	5 (14.7%)	5 (29.4%)	0 (0.0%)	10.80 (.013)
Other	3 (3.0%)	1 (8.3%)	0 (0.0%)	1 (5.9%)	1 (2.8%)	2.70 (.442)

*Note.* SD = standard deviation, OCD = obsessive–compulsive disorder, PTSD = post-traumatic stress disorder, and ADHD = attention-deficit hyperactivity disorder.

**Table 2:** Depression and substance use across sessions among completers (*N* = 91).

Substance/Scale	*N* (%)	Mean (SD), Range	*T* (*p*)
TLFB Total			
Session 1	51 (51.5%)	2.07 (3.56)	
Session 2	37 (40.7%)	1.60 (3.30)	
TLFB Alcohol			
Session 1	45 (45.5%)	0.82 (1.15), 0.57–1.06	
Session 2	30 (33.0%)	0.53 (1.01), 0.32–0.74	3.44 (<.001)
TLFB Cannabis			
Session 1	19 (20.2%)	0.82 (1.15)	
Session 2	13 (14.9%)	0.53 (1.01)	
TLFB Stimulants			
Session 1	6 (6.5%)	0.71 (1.80)	
Session 2	5 (5.9%)	0.60 (1.70)	
TLFB Hallucinogens			
Session 1	1 (1.1%)	0.11 (0.10)	
Session 2	1 (1.1%)	0.11 (0.10)	
TLFB Sedatives			
Session 1	0 (0.0%)	0.00 (0.00)	
Session 2	1 (1.2%)	0.01 (0.10)	
TLFB Other			
Session 1	4 (6.9%)	0.36 (1.45)	
Session 2	2 (3.0%)	0.19 (1.12)	
PHQ-9 Score			
Session 1		6.54 (4.82), 5.54–7.54	
Session 2		6.30 (4.83), 5.29–7.30	0.59 (.555)

*Note.* TLFB = timeline follow back. There was no reported use of cocaine, crack, opioids, heroin, benzodiazepines, or inhalants. Means for substance use variables represent the average number of days a week the substance was used.

**Table 3:** Engagement and alliance among completers (*N* = 91).

	Total *N* = 91	Depression Only *N* = 11	Substance Use Only *N* = 33	Co-Occurring *N* = 13	No Substance Use or Depression *N* = 34	Time	Group	Group × Time
	Mean (SD), Range					*F* (*p*)		
UES-SF Total								
Session 1	3.00 (0.63), 1.50–4.30	3.18 (0.72), 2.00–4.30	2.84 (0.58), 1.50–3.70	3.19 (0.81), 1.80–4.30	3.02 (0.55), 1.70–4.00	11.72 (<.001)	0.66 (.577)	1.47 (.228)
Session 2	3.23 (0.67), 1.58–4.58	3.23 (0.75), 1.58–4.17	3.18 (0.63), 1.67–4.25	3.32 (0.95), 1.67–4.58	3.25 (0.58), 1.83–4.33			
UES-SF Focus								
Session 1	2.50 (0.86), 1.00–4.67	2.70 (0.94), 1.33–4.00	2.38 (0.73), 1.00–4.00	2.95 (0.73), 1.67–4.00	2.37 (0.95), 1.00–4.67	0.03 (.861)	1.24 (.299)	0.54 (.658)
Session 2	2.56 (0.99), 1.00–4.67	2.73 (0.92), 1.00–4.00	2.56 (0.97), 1.00–4.33	2.77 (1.10), 1.00–4.00	2.42 (0.99), 1.00–4.67			
UES-SF Usability								
Session 1	4.14 (0.87), 1.00–5.00	3.95 (1.21), 1.00–5.00	3.98 (0.82), 3.00–5.00	4.15 (0.80), 3.00–5.00	4.34 (0.80), 3.00–5.00	4.41 (.0394)	0.66 (.581)	0.94 (.423)
Session 2	4.31 (0.75), 2.33–5.00	4.15 (0.69), 3.00–5.00	4.27 (0.78), 2.33–5.00	4.21 (0.81), 2.67–5.00	4.44 (0.72), 2.33–5.00			
UES-SF Aesthetic								
Session 1	2.37 (0.98), 1.00–4.33	2.88 (1.00), 1.00–4.00	2.07 (0.83), 1.00–3.67	2.64 (1.10), 1.00–4.00	2.39 (0.99), 1.00–4.33	0.03 (.858)	1.33 (.271)	3.37 (.022)
Session 2	2.49 (0.98), 1.00–5.00	2.36 (0.95), 1.00–4.00	2.33 (0.77), 1.00–3.67	2.77 (1.25), 1.00–4.67	2.58 (1.06), 1.00–5.00			
UES-SF Reward								
Session 1	3.56 (0.97), 1.00–5.00	3.59 (1.02), 2.00–5.00	3.53 (0.91), 1.00–5.00	3.42 (1.40), 1.00–5.00	3.63 (0.85), 2.00–5.00	0.25 (.617)	0.08 (.970)	0.24 (.865)
Session 2	3.58 (0.96), 1.00–5.00	3.67 (1.06), 1.33–5.00	3.56 (0.82), 2.00–4.67	3.54 (1.31), 1.00–5.00	3.58 (0.95), 1.00–5.00			
BR-WAI								
Session 1	15.80 (5.84), 6.00–30.00	18.27 (6.80), 8.00–27.00	15.55 (5.71), 6.00–30.00	16.54 (5.30), 7.00–25.00	14.97 (5.84), 7.00–30.00	0.72 (.397)	0.66 (.579)	0.54 (.656)
Session 2	16.56 (5.21), 6.00–29.00	17.45 (5.57), 8.00–26.00	16.42 (4.48), 6.00–26.00	17.15 (5.46), 7.00–25.00	16.18 (5.82), 6.00–29.00			
RITch^®^CBT Number of Sessions								
Session 1	4.31 (2.85), 0–10	5.27 (3.13), 2–10	4.48 (3.00), 0–10	4.69 (3.30), 2–10	3.68 (2.38), 0–10	0.35 (.554)	0.79 (.505)	0.42 (.740)
Session 2	4.27 (2.94), 0–10	4.36 (3.14), 0–10	4.58 (2.96), 0–10	4.38 (3.40), 1–10	3.91 (2.76), 0–10			
RITch^®^CBT Practice Total								
Session 1	N/A	N/A	N/A	N/A	N/A	N/A	1.91 (.138)	N/A
Session 2	3.10 (2.50), 0–10	5.00 (2.33), 1–8	2.68 (2.08), 0–8	2.75 (2.14), 0–5	3.04 (2.88), 0–10			
RITch^®^CBT Practice Helpful								
Session 1	N/A	N/A	N/A	N/A	N/A	N/A	1.39 (.255)	N/A
Session 2	4.13 (2.84), 0–10	6.00 (2.88), 1–10	3.91 (2.71), 0–9	3.58 (2.75), 0–10	4.00 (2.93), 0–8			

*Note.* SD = standard deviation, UES-SF = User Engagement Scale—Short Form, BR-WAI = Brief-Revised Working Alliance Inventory.

## Data Availability

The data presented in this study are available on request from the corresponding author due to privacy/intellectual property of the digital platform.

## References

[B1-behavsci-16-00719] Adams S. H., Knopf D. K., Park M. J. (2014). Prevalence and treatment of mental health and substance use problems in the early emerging adult years in the United States: Findings from the 2010 national survey on drug use and health. Emerging Adulthood.

[B2-behavsci-16-00719] Adams Z. W., Dellucci T. V., Agley J., Bixler K., Sullivan M., Hinckley J. D., Hulvershorn L. A. (2025). Estimated prevalence of substance use disorders among US adolescents and emerging adults by substance class, severity, and age, 2022. JAACAP Open.

[B3-behavsci-16-00719] Agrawal S., Sobell M. B., Sobell L. C., Belli R. F., Stafford F. P., Alwin D. F. (2009). The timeline followback: A scientifically and clinically useful tool for assessing substance use. Calendar and time diary methods in life course research.

[B4-behavsci-16-00719] Alhammad M., Aljedani R., Alsaleh M., Atyia N., Alsmakh M., Alfaraj A., Alkhunaizi A., Alwabari J., Alzaidi M. (2022). Family, individual, and other risk factors contributing to risk of substance abuse in young adults: A narrative review. Cureus.

[B5-behavsci-16-00719] American Addiction Centers (AAC) (2024). Alcohol and drug use among for young adults.

[B6-behavsci-16-00719] Andrade A. Q., Beleigoli A. M., Silva T. M., Diniz M. D. F. H., Ribeiro A. L. P. (2019). Exploring the user engagement scale short form as a determinant of adherence in digital health interventions. Studies in Health Technology and Informatics.

[B7-behavsci-16-00719] Andrews J. A., Westling E., Arnett J. J. (2016). Substance use in emerging adulthood. The Oxford handbook of emerging adulthood.

[B8-behavsci-16-00719] Arnett J. J., Žukauskienė R., Sugimura K. (2014). The new life stage of emerging adulthood at ages 18–29 years: Implications for mental health. The Lancet Psychiatry.

[B9-behavsci-16-00719] Babor T. F., McRee B. G., Kassebaum P. A., Grimaldi P. L., Ahmed K., Bray J., Galanter M., Saitz R. (2008). Screening, brief intervention, and referral to treatment (SBIRT): Toward a public health approach to the management of substance abuse. Alcohol/drug screening and brief intervention.

[B10-behavsci-16-00719] Benitez B., Frankforter T. L., Nich C., Kiluk B. D. (2023). The connection still matters: Therapeutic alliance with digital treatment for alcohol use disorder. Alcohol: Clinical and Experimental Research.

[B11-behavsci-16-00719] Bhattacherjee A., Premkumar G. (2004). Understanding changes in belief and attitude toward information technology usage: A theoretical model and longitudinal test. MIS Quarterly.

[B12-behavsci-16-00719] Bilek F., Çetik F. N., Türk M., Yılmaz E. (2026). Determination of psychosocial care competence, therapeutic alliance and burnout levels in nurses. Journal of Psychiatric Nursing.

[B13-behavsci-16-00719] Bondarchuk C. P., Magidson J., Mellins C., Lemon T. L., Rousseau E., Sindelo S., Medina-Marino A., Sibanda N., Butler L. M., Bekker L., Earnshaw V. A., Katz I. T. (2025). Maladaptive coping strategies mediate the relationship between depression and anxiety and moderate-to-heavy alcohol use in young South Africans with HIV. AIDS and Behavior.

[B14-behavsci-16-00719] Boness C. L., Votaw V. R., Schwebel F. J., Moniz-Lewis D. I. K., McHugh R. K., Witkiewitz K. (2023). An evaluation of cognitive behavioral therapy for substance use disorder: A systematic review and application of the society of clinical psychology criteria for empirically supported treatments. Clinical Psychology: A Publication of the Division of Clinical Psychology of the American Psychological Association.

[B15-behavsci-16-00719] Borges G., Walters E. E., Kessler R. C. (2000). Associations of substance use, abuse, and dependence with subsequent suicidal behavior. American Journal of Epidemiology.

[B16-behavsci-16-00719] Borghouts J., Eikey E., Mark G., De Leon C., Schueller S. M., Schneider M., Stadnick N., Zheng K., Mukamel D., Sorkin D. H. (2021). Barriers to and facilitators of user engagement with digital mental health interventions: Systematic review. Journal of Medical Internet Research.

[B17-behavsci-16-00719] Borgnolo L. J., McKenna S., Hickie I. B., Varidel M., Turner A., Gorban C., LaMonica H. M., Chong M. K., Capon W., Dimitropoulos G., Battisti R., Whitwell B., Hamilton B., Scott E. M., Iorfino F. (2025). Understanding engagement with digital mental health technology in mental health services: Multicenter observational study. Journal of Medical Internet Research.

[B18-behavsci-16-00719] Boumparis N., Schulte M. H., Riper H. (2019). Digital mental health for alcohol and substance use disorders. Current Treatment Options in Psychiatry.

[B19-behavsci-16-00719] Bowers E. M., Klimczak K. S., Aller T. B., Levin M. E. (2026). The paradox of choice: User preferences and completion rates in single-session vs. multi-session digital mental health interventions. Cognitive Behaviour Therapy.

[B20-behavsci-16-00719] Brody D. J., Hughes J. P. (2025). Depression prevalence in adolescents and adults: United States, August 2021–August 2023.

[B21-behavsci-16-00719] Carreiro S., Newcomb M., Leach R., Ostrowski S., Boudreaux E. D., Amante D. (2020). Current reporting of usability and impact of mHealth interventions for substance use disorder: A systematic review. Drug and Alcohol Dependence.

[B22-behavsci-16-00719] Leshner A. I., Scherer L. A., Committee on Mental Health, Substance Use, and Wellbeing in STEMM Undergraduate and Graduate Education, Board on Higher Education and Workforce, Board on Health Sciences Policy, Policy and Global Affairs, Health and Medicine Division, National Academies of Sciences, Engineering, and Medicine (2021). Mental health, substance use, and wellbeing in higher education: Supporting the whole student.

[B23-behavsci-16-00719] Crawford J., Collier E. S., Bendtsen M. (2024). Individualized treatment effects of a digital alcohol intervention and their associations with participant characteristics and engagement. Alcohol and Alcoholism.

[B24-behavsci-16-00719] Dalton K., Bishop L., Darcy S. (2021). Investigating interventions that lead to the highest treatment retention for emerging adults with substance use disorder: A systematic review. Addictive Behaviors.

[B25-behavsci-16-00719] Denison-Day J. L., Muir S., Newell C., Appleton K. M. (2023). The role of aesthetics in intentions to use digital health interventions. PLoS Digital Health.

[B26-behavsci-16-00719] Dhami P., Quilty L. C., Schwartzmann B., Uher R., Allen T. A., Kloiber S., Lam R. W., MacQueen G., Frey B. N., Milev R., Müller D. J., Rotzinger S., Kennedy S. H., Farzan F. (2022). Alterations in the neural correlates of affective inhibitory control following cognitive behavioral therapy for depression: A Canadian biomarker integration network for depression (CAN-BIND) study. Journal of Affective Disorders Reports.

[B27-behavsci-16-00719] Dillon P. J., Kedia S. K., Isehunwa O. O., Sharma M. (2020). Motivations for treatment engagement in a residential substance use disorder treatment program: A qualitative study. Substance Abuse: Research and Treatment.

[B28-behavsci-16-00719] Easton C. J., Berbary C. M., Crane C. A. (2018a). Avatar and technology assisted platforms in the treatment of co-occurring addiction and IPV among male offenders. Advances in Dual Diagnosis.

[B29-behavsci-16-00719] Easton C. J., Crane C., Berbary C., Sangiorgio C., Kalahasthi P., Toole T. (2020). Use of an interactive & customizable RITch^®^-CBT avatar platform to treat clients in the healthcare field *[Symposium presentation]*. Frameless XR Symposium.

[B30-behavsci-16-00719] Easton C. J., Crane C. A., Mandel D. (2018b). A randomized controlled trial assessing the efficacy of cognitive behavioral therapy for substance-dependent domestic violence offenders: An integrated substance abuse-domestic violence treatment approach (SADV). Journal of Marital and Family Therapy.

[B31-behavsci-16-00719] Farhoudian A., Razaghi E., Hooshyari Z., Noroozi A., Pilevari A., Mokri A., Mohammadi M. R., Malekinejad M. (2022). Barriers and facilitators to substance use disorder treatment: An overview of systematic reviews. Substance Abuse: Research and Treatment.

[B32-behavsci-16-00719] Gasa H. P., Mkhize S., Shumba K., Cinini S. F., Gopal N. D. (2022). Risk factors of substance abuse among university students: An exploratory study. International Journal of Criminology.

[B33-behavsci-16-00719] Ghosh A., Singh P., Das N., Pandit P. M., Das S., Sarkar S. (2022). Efficacy of brief intervention for harmful and hazardous alcohol use: A systematic review and meta-analysis of studies from low middle-income countries. Addiction.

[B34-behavsci-16-00719] Gidhagen Y., Holmqvist R., Philips B., Falkenström F. (2021). The role of the working alliance in psychological treatment of substance use disorder outpatients. Psychotherapy Research.

[B35-behavsci-16-00719] Gilchrist G., Dheensa S., Johnson A., Henderson J., Radcliffe P., Dwyer G., Turner R., Thomson K., Papastavrou Brooks C., Love B., Zenasni Z., Berbary C., Carter B., Parrott S., Li J., Easton C., Bergman C., Feder G., Gilchrist E. (2024). Adapting the ADVANCE group program for digitally-supported delivery to reduce intimate partner violence by men in substance use treatment: A feasibility study. Frontiers in Psychiatry.

[B36-behavsci-16-00719] Goodwin R. D., Dierker L. C., Wu M., Galea S., Hoven C. W., Weinberger A. H. (2022). Trends in US depression prevalence from 2015 to 2020: The widening treatment gap. American Journal of Preventive Medicine.

[B37-behavsci-16-00719] Graham A. K., Kwasny M. J., Lattie E. G., Greene C. J., Gupta N. V., Reddy M., Mohr D. C. (2021). Targeting subjective engagement in experimental therapeutics for digital mental health interventions. Internet Interventions.

[B38-behavsci-16-00719] Graupensperger S., Janson M. A., Fairlie A. M., Larimer M. E., Lee C. M. (2025). Depressive symptoms and substance use: Longitudinal examination of alcohol and cannabis coping mechanisms in young adults. Prevention Science.

[B39-behavsci-16-00719] Grigoriu L. E., Benga O. (2025). Adolescent and young adult substance use. A theoretical review of internal and external factors. Studia Universitatis Babes-Bolyai, Psychologia-Paedagogia.

[B40-behavsci-16-00719] Günther F., Yau C., Elison-Davies S., Wong D. (2023). On the difficulty of predicting engagement with digital health for substance use. Caring is sharing–exploiting the value in data for health and innovation.

[B41-behavsci-16-00719] Hadland S. E., Yule A. M., Levy S. J., Hallett E., Silverstein M., Bagley S. M. (2021). Evidence-based treatment of young adults with substance use disorders. Pediatrics.

[B42-behavsci-16-00719] Hampton J., Mugambi P., Caggiano E., Eugene R., Valente A., Taylor M., Carreiro S. (2024). Closing the digital divide in interventions for substance use disorder. Journal of Psychiatry and Brain Science.

[B43-behavsci-16-00719] Hersoug A. G., Høglend P., Monsen J. T., Havik O. E. (2001). Quality of working alliance in psychotherapy: Therapist variables and patient/therapist similarity as predictors. The Journal of Psychotherapy Practice and Research.

[B44-behavsci-16-00719] Hill K. G., White H. R., Chung I. J., Hawkins J. D., Catalano R. F. (2000). Early adult outcomes of adolescent binge drinking: Person-and variable-centered analyses of binge drinking trajectories. Alcoholism: Clinical and Experimental Research.

[B45-behavsci-16-00719] Holm A. J., Hausman H., Rhodes M. G. (2022). Study strategies and “study drugs”: Investigating the relationship between college students’ study behaviors and prescription stimulant misuse. Journal of American College Health.

[B46-behavsci-16-00719] Hunt G. E., Malhi G. S., Lai H. M. X., Cleary M. (2020). Prevalence of comorbid substance use in major depressive disorder in community and clinical settings, 1990–2019: Systematic review and meta-analysis. Journal of Affective Disorders.

[B47-behavsci-16-00719] Iacono W. G., Malone S. M., McGue M. (2008). Behavioral disinhibition and the development of early-onset addiction: Common and specific influences. Annual Review of Clinical Psychology.

[B48-behavsci-16-00719] IBM Corp. (2024). IBM statistics for windows (version 31.0) *[Computer software]*.

[B49-behavsci-16-00719] Jonathan G. K., Guo Q., Arcese H., Evins A. E., Wilhelm S. (2025). Digital integrated interventions for comorbid depression and substance use disorder: Narrative review and content analysis. JMIR Mental Health.

[B50-behavsci-16-00719] Jones A. A., Hard G., Gray J., Apsley H. B., Santos-Lozada A. R. (2023). The role of substance use disorders on suicidal ideation, planning, and attempts: A nationally representative study of adolescents and adults in the United States, 2020. Substance Abuse: Research and Treatment.

[B51-behavsci-16-00719] Jurewicz I. (2015). Mental health in young adults and adolescents—Supporting general physicians to provide holistic care. Clinical Medicine.

[B52-behavsci-16-00719] Kessler R. C., Berglund P., Demler O., Jin R., Merikangas K. R., Walters E. E. (2005). Lifetime prevalence and age-of-onset distributions of DSM-IV disorders in the national comorbidity survey replication. Archives of General Psychiatry.

[B53-behavsci-16-00719] Kroenke K., Spitzer R. L., Williams J. B. (2001). The PHQ-9: Validity of a brief depression severity measure. Journal of General Internal Medicine.

[B54-behavsci-16-00719] Lakens D. (2022). Sample size justification. Collabra: Psychology.

[B55-behavsci-16-00719] Lalmas M., O’Brien H., Yom-Tov E. (2022). Measuring user engagement.

[B56-behavsci-16-00719] Lijffijt M., Hu K., Swann A. C. (2014). Stress modulates illness-course of substance use disorders: A translational review. Frontiers in Psychiatry.

[B57-behavsci-16-00719] Lipschitz J. M., Pike C. K., Hogan T. P., Murphy S. A., Burdick K. E. (2023). The engagement problem: A review of engagement with digital mental health interventions and recommendations for a path forward. Current Treatment Options in Psychiatry.

[B58-behavsci-16-00719] Lu W., Bessaha M., Muñoz-Laboy M. (2022). Examination of young US adults’ reasons for not seeking mental health care for depression, 2011–2019. JAMA Network Open.

[B59-behavsci-16-00719] Luderer H. F., Campbell A. N., Nunes E. V., Enman N. M., Xiong X., Gerwien R., Maricich Y. A. (2022). Engagement patterns with a digital therapeutic for substance use disorders: Correlations with abstinence outcomes. Journal of Substance Abuse Treatment.

[B60-behavsci-16-00719] Magill M., Helminen E. C., Lynch-Gadaleta B., Allen K., Kiluk B. D., Ray L. A. (2025). Cognitive-behavioral interventions for co-occurring substance use and mental health disorders. Drug and Alcohol Dependence.

[B61-behavsci-16-00719] Magill M., Kiluk B. D., Ray L. A. (2023). Efficacy of cognitive behavioral therapy for alcohol and other drug use disorders: Is a one-size-fits-all approach appropriate?. Substance Abuse and Rehabilitation.

[B62-behavsci-16-00719] Mallinckrodt B., Tekie Y. T. (2015). Revision of the working alliance inventory and development of a brief revised version guided by item response theory.

[B63-behavsci-16-00719] Martens T. (2022). Relationship between drug addiction, alcoholism, and violence.

[B64-behavsci-16-00719] McCann N. C., Yan S., McMahan V. M., Pope E., Rolles A., Brennan S., Marti X. L., Kosakowski S., Coffin P. O., Walley A. Y. (2025). Test-retest reliability of a timeline follow-back method to assess opioid use and treatment. Journal of Addiction Medicine.

[B65-behavsci-16-00719] McConaha C. D., McCabe B. E., Falcon A. L. (2024). Anxiety, depression, coping, alcohol use and consequences in young adult college students. Substance Use & Misuse.

[B66-behavsci-16-00719] McGovern M. P., Dunn J., Bonnell L. N., Leibowitz G., Waddell E., Rose G., Littenberg B. (2023). The association between depression and substance use among primary care patients with comorbid medical and behavioral health conditions. Journal of Primary Care & Community Health.

[B67-behavsci-16-00719] McHugh R. K., Hearon B. A., Otto M. W. (2010). Cognitive behavioral therapy for substance use disorders. The Psychiatric Clinics of North America.

[B68-behavsci-16-00719] Moilanen J., Visuri A., Suryanarayana S. A., Alorwu A., Yatani K., Hosio S. (2022). Measuring the effect of mental health chatbot personality on user engagement. Proceedings of the 21st international conference on mobile and ubiquitous multimedia, Lisbon, Portugal.

[B69-behavsci-16-00719] Moriuchi E., Berbary C., Easton C. (2023). Looking through the lenses of a patient: An empirical study on the factors affecting patients’ intention to use avatar-assisted therapy. Journal of Technology in Behavioral Science.

[B70-behavsci-16-00719] Munson M. R., Jaccard J., Moore K. L., Rodwin A. H., Shimizu R., Cole A. R., Scott L. D. J., Narendorf S. C., Davis M., Gilmer T., Stanhope V. (2022). Impact of a brief intervention to improve engagement in a recovery program for young adults with serious mental illness. Schizophrenia Research.

[B71-behavsci-16-00719] National Institute on Drug Abuse (NIDA) (2023). What is the scope of prescription drug misuse in the United States?.

[B72-behavsci-16-00719] Nordheim K., Walderhaug E., Alstadius S., Kern-Godal A., Arnevik E., Duckert F. (2018). Young adults’ reasons for dropout from residential substance use disorder treatment. Qualitative Social Work.

[B73-behavsci-16-00719] O’Brien H. L., Cairns P., Hall M. (2018). A practical approach to measuring user engagement with the refined user engagement scale (UES) and new UES short form. International Journal of Human-Computer Studies.

[B74-behavsci-16-00719] Oesterle T. S., Bormann N. L. (2026). Digital therapies for substance use disorders: Recent advances and engagement strategies. Substance Abuse and Rehabilitation.

[B75-behavsci-16-00719] Pardini S., Gabrielli S., Dianti M., Novara C., Zucco G. M., Mich O., Forti S. (2022). The role of personalization in the user experience, preferences and engagement with virtual reality environments for relaxation. International Journal of Environmental Research and Public Health.

[B76-behavsci-16-00719] Patrick M. E., Miech R. A., Johnston L. D., O’Malley P. M. (2025). Monitoring the future panel study annual report: National data on substance use among adults ages 19 to 65, 1976–2024 *[Monitoring the Future Monograph Series]*.

[B77-behavsci-16-00719] Pitts B. H., Sheeder J., Sigel E., Love-Osborne K., Woods J. (2023). Informing use of the patient health questionnaire-2 to detect moderate or greater depression symptoms in adolescents and young adults in outpatient primary care. Journal of Adolescent Health.

[B78-behavsci-16-00719] Posit Team (2025). RStudio: Integrated development environment for R.

[B79-behavsci-16-00719] R Core Team (2026). R: A language and environment for statistical computing.

[B80-behavsci-16-00719] Reifman A., Niehuis S. (2023). Extending the five psychological features of emerging adulthood into established adulthood. Journal of Adult Development.

[B81-behavsci-16-00719] Reinert M., Fritze D., Nguyen T. (2024). The state of mental health in America 2024.

[B82-behavsci-16-00719] Salaheddin K., Mason B. (2016). Identifying barriers to mental health help-seeking among young adults in the UK: A cross-sectional survey. The British Journal of General Practice: The Journal of the Royal College of General Practitioners.

[B83-behavsci-16-00719] Saleem M., Kühne L., De Santis K. K., Christianson L., Brand T., Busse H. (2021). Understanding engagement strategies in digital interventions for mental health promotion: Scoping review. JMIR Mental Health.

[B84-behavsci-16-00719] Schwartz S. J., Zamboanga B. L., Luyckx K., Meca A., Ritchie R. A. (2013). Identity in emerging adulthood: Reviewing the field and looking forward. Emerging Adulthood.

[B85-behavsci-16-00719] Sheidow A. J., McCart M., Zajac K., Davis M. (2012). Prevalence and impact of substance use among emerging adults with serious mental health conditions. Psychiatric Rehabilitation Journal.

[B86-behavsci-16-00719] Silvers J. A., Peris T. S. (2023). Research review: The neuroscience of emerging adulthood–reward, ambiguity, and social support as building blocks of mental health. Journal of Child Psychology and Psychiatry.

[B87-behavsci-16-00719] Silverstein M., Hadland S. E., Hallett E., Botticelli M. (2021). Principles of care for young adults with co-occurring psychiatric and substance use disorders. Pediatrics.

[B88-behavsci-16-00719] Sinha R. (2024). Stress and substance use disorders: Risk, relapse, and treatment outcomes. The Journal of Clinical Investigation.

[B89-behavsci-16-00719] Smith K. A., Ward T., Lambe S., Ostinelli E. G., Blease C., Gant T., Gold S. M., Holmes E. A., Paccoud I., Vinnokova A., Klucken J., Uhlhaas P. J., Sanchez C. G., Haining K., Böge K., Lahutina S., Tomelleri L., Ryan S., Torous J., Cipriani A. (2025). Engagement and attrition in digital mental health: Current challenges and potential solutions. NPJ Digital Medicine.

[B90-behavsci-16-00719] Solmi M., Radua J., Olivola M., Croce E., Soardo L., Salazar de Pablo G., Il Shin J., Kirkbride J. B., Jones P., Kim J. H., Kim J. Y., Carvalho A. F., Seeman M. V., Correll C. U., Fusar-Poli P. (2022). Age at onset of mental disorders worldwide: Large-scale meta-analysis of 192 epidemiological studies. Molecular Psychiatry.

[B91-behavsci-16-00719] Sonderegger A., Sauer J. (2010). The influence of design aesthetics in usability testing: Effects on user performance and perceived usability. Applied Ergonomics.

[B92-behavsci-16-00719] Spek V., Cuijpers P. I. M., Nyklíček I., Riper H., Keyzer J., Pop V. (2007). Internet-based cognitive behaviour therapy for symptoms of depression and anxiety: A meta-analysis. Psychological Medicine.

[B93-behavsci-16-00719] Steinhoff A., Shanahan L., Bechtiger L., Zimmermann J., Ribeaud D., Eisner M. P., Baumgartner M. R., Quednow B. B. (2023). When substance use is underreported: Comparing self-reports and hair toxicology in an urban cohort of young adults. Journal of the American Academy of Child & Adolescent Psychiatry.

[B94-behavsci-16-00719] Stone A. L., Becker L. G., Huber A. M., Catalano R. F. (2012). Review of risk and protective factors of substance use and problem use in emerging adulthood. Addictive Behaviors.

[B95-behavsci-16-00719] Substance Abuse and Mental Health Services Administration (SAMHSA) (2019). Substance misuse prevention for young adults.

[B96-behavsci-16-00719] Substance Abuse and Mental Health Services Administration (SAMHSA) (2022). Highlights for the 2022 national survey on drug use and health.

[B97-behavsci-16-00719] Substance Abuse and Mental Health Services Administration (SAMHSA) (2023). National survey on drug use and health.

[B98-behavsci-16-00719] Substance Abuse and Mental Health Services Administration (SAMHSA) (2024). 2023 Companion infographic report: Results from the 2021, 2022, and 2023 national surveys on drug use and health *(SAMHSA Publication No. PEP24-07-020)*.

[B99-behavsci-16-00719] Substance Abuse and Mental Health Services Administration (SAMHSA) (2025). Key substance use and mental health indicators in the United States: Results from the 2024 national survey on drug use and health *(HHS Publication No. PEP25-07-007, NSDUH Series H-60)*.

[B100-behavsci-16-00719] Urbanoski K. A., Kelly J. F., Hoeppner B. B., Slaymaker V. (2012). The role of therapeutic alliance in substance use disorder treatment for young adults. Journal of Substance Abuse Treatment.

[B101-behavsci-16-00719] van Benthem P., van der Lans R. M., Lamers A., Blanken P., Spijkerman R., Vermeiren R. R. J. M., Hendriks V. M. (2024). The working alliance inventory—Short version: Psychometric properties of the patient and therapist form in youth mental health and addiction care. BMC Psychology.

[B102-behavsci-16-00719] Vereschagin M., Wang A. Y., Richardson C. G., Xie H., Munthali R. J., Hudec K. L., Leung C., Wojcik K. D., Munro L., Halli P., Kessler R. C., Vigo D. V. (2024). Effectiveness of the minder mobile mental health and substance use intervention for university students: Randomized controlled trial. Journal of Medical Internet Research.

[B103-behavsci-16-00719] Vik P. W., Cellucci T., Jarchow A., Hedt J. (2004). Cognitive impairment in substance abuse. Psychiatric Clinics.

[B104-behavsci-16-00719] Viner J., Tanner J. L. (2009). Psychiatric disorders in emerging adulthood. Yellowbrick Journal of Emerging Adulthood.

[B105-behavsci-16-00719] Volkow N. D., Blanco C. (2023). Substance use disorders: A comprehensive update of classification, epidemiology, neurobiology, clinical aspects, treatment and prevention. World Psychiatry.

[B106-behavsci-16-00719] Walczak A. (2023). What does it mean to be an adult? Adulthood markers in the perspective of emerging adults. Emerging Adulthood.

[B107-behavsci-16-00719] Wanniarachchi V. U., Greenhalgh C., Choi A., Warren J. R. (2025). Personalization variables in digital mental health interventions for depression and anxiety in adolescents and youth: A scoping review. Frontiers in Digital Health.

[B108-behavsci-16-00719] Williams A. D., Thompson J., Andrews G. (2013). The impact of psychological distress tolerance in the treatment of depression. Behaviour Research and Therapy.

[B109-behavsci-16-00719] Zainal N. H., Wang V., Garthwaite B., Curtiss J. E. (2026). What factors are related to engagement with digital mental health interventions (DMHIs)? A meta-analysis of 117 trials. Health Psychology Review.

